# Unfractionated Heparin in SARS-CoV-2 Pneumonia: Ischemic Stroke Case Report

**DOI:** 10.3389/fneur.2020.573356

**Published:** 2020-09-25

**Authors:** Aslan Efendizade, Adam A. Dmytriw, Kevin Hewitt, Gwynivere A. Davies

**Affiliations:** ^1^Department of Diagnostic Radiology, State University of New York (SUNY) Downstate Medical Center, University Hospital Brooklyn, Brooklyn, NY, United States; ^2^Department of Medical Imaging, University of Toronto, Toronto, ON, Canada; ^3^Department of Neuroradiology & Neurointervention, Brigham and Women's Hospital, Boston, MA, United States; ^4^Department of Oncology, Juravinski Cancer Centre, McMaster University, Hamilton, ON, Canada

**Keywords:** COVID-19, stroke, anticoagulation, heparin, thromboembolism

## Abstract

Thromboembolism is a known phenomenon in patients with Coronavirus disease 2019 (COVID-19). Recent investigations have revealed that a significant proportion of those hospitalized with severe COVID-19 demonstrate clinical and laboratory markers compatible with hypercoagulability, which is differentiated from disseminated intravascular coagulation (DIC), termed COVID-associated coagulopathy. Additionally, there is increasing concern for development of acute ischemic stroke because of this hypercoagulable state. We present a patient with COVID-19 pneumonia who was managed with unfractionated heparin (UFH) infusion and developed a large ischemic infarct shortly after cessation of the infusion. In retrospect, the patient's coagulation parameters were consistent with overt DIC, although some of these parameters are easily masked by the effects of UFH. These findings emphasize the importance of anticoagulation as well as its careful discontinuation, as failure to do so may result in a significant thromboembolic event.

## Introduction

Preliminary observations of Coronavirus disease 2019 (COVID-19) were consistent with hypoxemic respiratory failure from acute respiratory distress syndrome (ARDS) (1, 2). However, recent investigations have led researchers to question whether the predominant cause of respiratory failure is vascular, with development of microthrombi and pulmonary vasodilatation (3). This is especially relevant given the incidence of venous thromboembolism (VTE) and risk of disseminated intravascular coagulation (DIC) in patients with COVID-19 (2, 4, 5).

An analysis of 1,026 admitted Chinese patients demonstrated that 40% were considered high risk of developing VTE, with many at high risk for bleeding and death, suggesting the need for careful prophylaxis (6). Cui et al. (7) reported a single-center experience of 81 patients in ICU with severe COVID-19 infection who demonstrated a 25% VTE rate [though these patients did not receive prophylactic anticoagulation (7)]. Patients with VTE were older, had significantly lower lymphocyte counts, higher D-dimer values, and prolonged activated partial thromboplastin times (aPTT).

Many patients with sepsis demonstrate deranged coagulation factors. DIC is characterized by dysregulation of coagulation and fibrinolysis, resulting in widespread thrombosis and hemorrhage. Several societies have formulated diagnostic criteria for DIC, such as the International Society of Thrombosis and Hemostasis (ISTH), which tends to classify cases into overt or non-overt DIC. The classification system assigns points (0–3) based on values associated with each parameter—platelet count, fibrin-related markers, prothrombin time (PT), and fibrinogen—and a score ≥5 is compatible with overt DIC (8). One study of 183 hospitalized patients with COVID-19 found that 71.4% of non-survivors (and 0.6% of survivors) met the criteria for overt DIC according to ISTH criteria (9). Subgroup analysis of 99 patients (with high sepsis-induced coagulopathy scores or D-dimer values) who received prophylactic anticoagulation demonstrated significantly reduced mortality, which led some institutions to adopt intermediate or therapeutic anticoagulation for severe cases of COVID-19. How best to dose, time, and discontinue anticoagulation remains to be determined.

## Case Description

**Figure 1:**
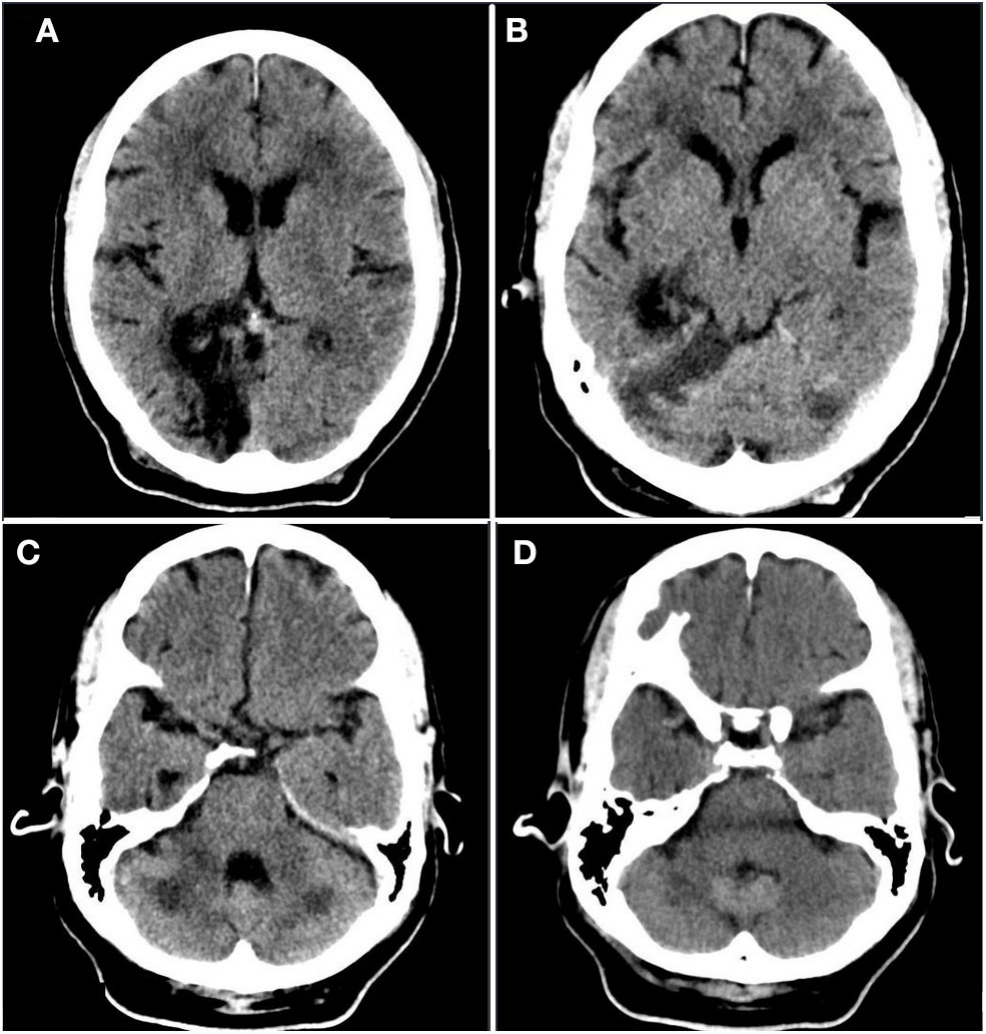
Prior infarct. Rostral to caudal **(A–D)** non-contrast computed tomographic axial slices of the head performed 2 years prior to current admission demonstrates hypoattenuation and parenchymal volume loss in the right parasagittal occipital lobe compatible with an old posterior cerebral artery territory infarct, as well as acute infarct in the territory of the bilateral superior cerebellar arteries.

A 56-year-old Haitian man with past medical history significant for hypertension, diabetes mellitus, seizure disorder, and prior cerebrovascular accident (CVA) (Figure 1) of unknown etiology with residual dysarthria and gait abnormality presented with worsening dry cough and dyspnea on exertion. He denied other respiratory symptoms, fevers, myalgia, sick contacts, and recent travel, as well as novel neurological symptoms. Home medications included amlodipine, aspirin, clopidogrel, atorvastatin, metformin, and levetiracetam.

In the emergency department, the patient was afebrile with an oxygen saturation of 50% on room air, improving to 91% on bilevel positive airway pressure. Physical examination revealed crackles within the lung bases, and the patient was unable to speak in full sentences. Additionally, it was noted that he was not oriented to time or person, nor was he cooperative with a neurological exam, although novel focal neurological deficits were not observed. Relevant admission laboratory markers are summarized (Table 1). Electrocardiogram was unremarkable, and chest radiography revealed bilateral multifocal airspace opacities. SARS-CoV-2 polymerase chain reaction by nasopharyngeal sampling was positive, and the patient was started on hydroxychloroquine, ceftriaxone, and azithromycin in addition to standard supportive care.

**Table 1:** Relevant admission laboratory markers.

**Marker**	**Value**	**Reference range**
Lymphocyte count ( ×10^3^/μl)	0.5	0.9–2.9
Creatinine (mg/dl)	2.0	0.7–1.3
AST (U/L)	153	13–19
ALT (U/L)	81	7–52
CRP (mg/L)	304	0–8
Ferritin (μg/L)	2,495	16–294
LDH (U/L)	1,046	140–271
Procalcitonin (ng/ml)	0.58	0.00–0.10

*Additional labs seen in “Day 1” of Table 2. AST, aspartate transaminase; ALT, alanine transaminase; CRP, C-reactive protein; LDH, lactate dehydrogenase*.

On the second day of admission, the patient's hypoxemia worsened, requiring intubation and mechanical ventilation with a fraction of inspired oxygen of 0.50. Further D-Dimer elevation (>4,000 ng/ml FEU) led to initiation of therapeutic unfractionated heparin (UFH). Based on management criteria (10), UFH was selected for concomitant acute renal failure (ARF).

**Figure 2:**
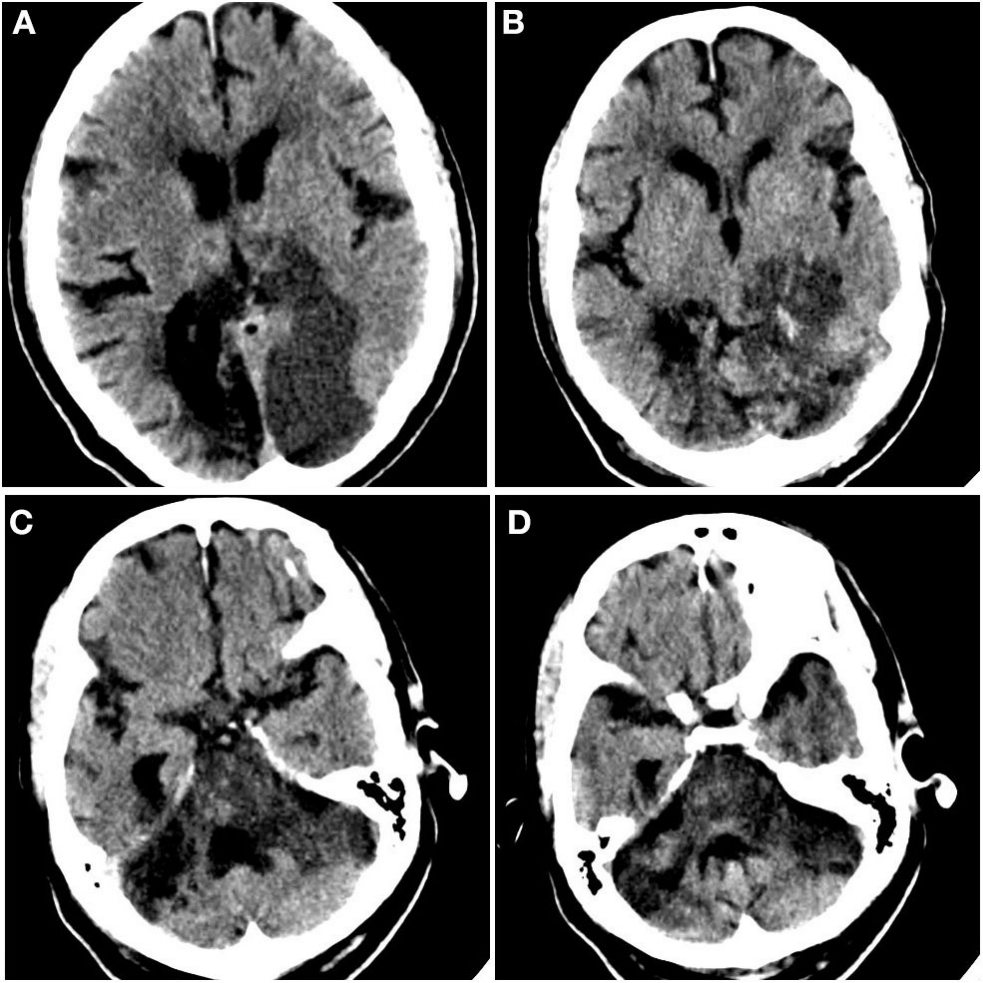
Acute ischemic infarct. Rostral to caudal **(A–D)** non-contrast computed tomographic axial slices of the head performed on this admission demonstrates loss of gray–white differentiation in the parasagittal left occipital lobe and sulcal effacement compatible with recent ischemia, as well as diffuse brainstem edema. Additional surrounding punctate foci of hyperattenuation suggest petechial hemorrhage.

On day 5, UFH was held secondary to elevation of aPTT (Table 2). Approximately 2 h after, the patient developed a non-reactive left pupil followed by myoclonic head movements 6 h after that. Given that the patient was intubated and under sedation, neurological examination was limited, and this was initially managed as seizure activity given the patient's history. However, he was non-responsive to antiseizure medications. Subsequent neurological evaluation revealed a left fixed pupil (4 mm), absent corneal and vestibulo-ocular reflexes, and no response to painful stimulation. The patient then underwent computed tomography (CT) of the head, which revealed infarcts within the parasagittal left occipital lobe and brainstem (Figure 2). Although advanced imaging was not performed, on coronal and sagittal reconstructions of the non-contrast head CT, a dense vessel sign was observed extending from the mid basilar into the left posterior cerebral artery. It should be noted that on the day prior, a bedside transthoracic echocardiogram was unremarkable, and electrocardiogram did not reveal an arrhythmia. Unfortunately, despite supportive care, the patient passed away.

**Table 2:** Timeline of coagulation parameters.

**Marker**	**Day 1**	**Day 2**	**Day 3**	**Day 4**	**Day 5**	**Day 6**	**Day 7**
Anticoagulation	SubQ heparin	SubQ heparin	Heparin drip	Heparin drip	Drip held <8 A.M	–	–
D-Dimer^a^ (ng/ml FEU)	1,704	–	>4,000	–	–	–	–
PT (s)	–	–	13.7	–	–	13.5	16.3
aPTT (s)	40	–	–	90.3	86.9	27.8	37.5
Platelets ( ×10^3^/μl)	169	200	219	238	220—Large	248—Large	219—Large

*On days 1 to 3, the patient received prophylactic subcutaneous heparin 5,000 units every 8 h. On day 3, the patient was transitioned to heparin drip at 1,000 units/h with target aPTT of 60–80 s. On the night of day 4, the patient had equal and reactive pupils. Heparin drip was held before 8 A.M on day 5, and (at 10 A.M) the patient's left pupil became non-reactive and at ~4 P.M displayed myoclonic head movements. PT, prothrombin time; aPTT, activated partial thromboplastin time; Large, large platelets seen on smear*.

a *D-Dimer reference level 500 ng/ml or less of fibrinogen equivalent units (FEU)*.

## Discussion

### Disseminated Intravascular Coagulation

Suggested management of COVID-19 involves monitoring D-Dimer, prothrombin time (PT), international normalized ratio, aPTT, fibrinogen, and platelet counts (11), consistent with sepsis guidelines. The incidence of DIC in COVID-19 patients varies widely by severity of presentation but can be seen in >2/3 of patients who die from severe disease (5). Non-survivors also had significantly higher fibrin degradation product levels and prolonged PT and aPTT values at admission. Interestingly, a meta-analysis of nine studies investigating COVID-19 demonstrated significantly higher values of PT and D-dimer for severe vs. mild cases, but no difference in aPTT or platelet count was observed (12). Relatively mild thrombocytopenia and the disproportionate increase in PT vs. aPTT has led to adoption of the term COVID-associated coagulopathy (CAC).

Typical management strategy for sepsis-associated DIC involves treatment of the underlying infection. Unfortunately, there is no known effective treatment for COVID-19 infection. In DIC with a thrombotic phenotype, therapeutic doses of heparin have been suggested with one randomized controlled trial (RCT) demonstrating superior efficacy of LMWH compared to UFH (13). However, prophylaxis using LMWH, UFH, or mechanical thromboprophylaxis remains the standard of care in most patients with DIC.

### Anticoagulation

Several medical centers have published guidelines on prophylactic or therapeutic anticoagulation for COVID-19 patients based on D-Dimer levels and VTE occurrence (10, 11). At our Brooklyn center, both prophylactic and therapeutic anticoagulation is often achieved with apixaban or enoxaparin. UFH infusion is reserved for those with ARF. The Journal of the American College of Cardiology released guidelines addressing the management of COVID-19 associated thromboembolic events (4), identifying additional risk factors including critical illness, prolonged hospitalization and intubation, immobility, and use of investigational therapies. Troponin elevations in COVID-19 patients may therefore not be a direct result of infection but rather inflammation leading to plaque rupture or microthrombi from cytokine storm. For anticoagulation, they recommend prophylactic dosing, with an emphasis on LMWH to reduce health care worker exposure from blood draws or medication management.

### Potential Role of UFH in DIC and COVID-19

If the decision is made to initiate UFH, the subsequent diagnosis of DIC may be delayed for various reasons including reduced platelet consumption, attribution of PT elevation to UFH (14), reduced D-Dimer, and fibrinogen variability (an acute phase reactant). Therefore, use of UFH may lead to delayed recognition of progressive coagulopathy, potentially increasing their risk for adverse consequences. Utilizing the ISTH criteria for DIC, we can retrospectively calculate a score of 5 based on PT and D-Dimer values on the third day of admission (Table 2), which is compatible with overt DIC.

Additionally, precautions must be used for timing of medications as previous studies have shown rebound coagulopathy with discontinuation of both UFH and LMWH. In one RCT of patients with acute coronary syndrome, plasma prothrombin fragment and thrombin–antithrombin levels post-discontinuation exceeded levels both during and prior to treatment (15). While this change occurred faster in UFH, the peak levels after LMWH discontinuation were higher, suggesting that both can result in reactivation of the coagulation system, causing thrombus growth, and platelet recruitment. Given the long clinical course of COVID-19 and lack of definitive treatment, patients are thus at higher risk of thrombosis with sudden discontinuation of anticoagulation, which should be discouraged except in the case of clinically relevant bleeding. UFH nomograms should be re-evaluated considering this specific CAC phenotype, whereby preserved platelet counts may predispose to thrombotic events. In contrast, though a study of 221 patients with COVID-19 demonstrated 13 cases of cerebrovascular events, none of these are reported to have occurred in the setting of anticoagulation discontinuation or adjustment (16).

Once a thrombus has formed, the use of thrombolytics, such as tissue plasminogen activator (tPA), may be warranted if timely revascularization would relay a mortality benefit (with massive pulmonary embolism or ischemic stroke). Addressing this, a case series from Poor et al. in which five critically ill COVID-19 patients with ventilator-dependent respiratory failure demonstrated improvement with systemic tPA (3), suggested the presence of microvascular thrombi that did not respond to prophylactic or therapeutic anticoagulation. In our case, given the delay in symptom recognition and diagnosis of ischemic infarct, the patient was not a candidate for administration of thrombolytics. Furthermore, the extent of edema seen on non-contrast head CT suggests that the majority (if not entirety) of the involved vascular territory was infarcted, precluding endovascular thrombectomy.

The relationship between COVID-19 and ischemic stroke is still under investigation. Recently, two multi-center studies have been conducted to investigate the incidence and shed light on the possible etiology. In one study comparing the incidence of stroke in hospitalized or emergency department visits, the authors report an incidence of 1.6% in COVID-19 vs. 0.2% in patients with influenza (17). Another study performed in close geographic proximity to our institution demonstrated 0.9% incidence of imaging proven stroke in patients hospitalized with COVID-19 (18). Of those cases, the authors report that a majority (65.6%) were cryptogenic in etiology and that the observed cases were more likely to be in younger men as compared to historical controls.

## Limitations

Given the tumultuous and resource-strained context during this incident case, there are several limitations. No advanced neuroimaging or Doppler studies were performed as part of the stroke workup. Given the territory of involved brain parenchyma, the location of prior CVA, and the dense vessel seen on head CT, thromboembolism is a possible etiology. However, it should be noted that prior imaging (Figure 1) demonstrates the presence of a remote and acute infarct, suggesting that the new infarct (Figure 2) likely occurred in the setting of vertebrobasilar disease. With respect to underlying vertebrobasilar disease, blood pressure monitoring in the days leading up to the stroke suggests that a hypotensive episode in the setting of vertebrobasilar disease is less likely as the patient's lowest recorded mean arterial pressure was 78 mmHg.

The period during this admission was quite worrisome for our hospital, such that presence in the patients' rooms and high-risk interactions were limited and may have contributed to a delay in timely and thorough neurological investigation. Furthermore, in the days leading up to the stroke, the patient was intubated and under sedation such that typical manifestations of an acute stroke were not displayed. Thus, it may be the case that the ischemic event occurred before interruption of UFH. Furthermore, additional laboratory investigations were not performed to exclude other etiologies of coagulopathy, such as antiphospholipid antibodies. Finally, venous duplex studies of the extremities or CT pulmonary angiography were not performed to suggest concomitant thromboembolic events.

## Conclusion

In conclusion, we present a patient with COVID-19 pneumonia who was initiated on UFH for elevated coagulation parameters and subsequently developed neurological symptoms after 2-h UFH interruption, which may have been a sequela of acute ischemic infarct. Additionally, the patient's retrospective ISTH score was compatible with overt DIC, although diagnosis may have been delayed due to UFH effects. This supports the finding of increasing thrombotic risk with COVID-19 that can occur concurrently with an unusual DIC phenotype, outlining the importance of prophylaxis and careful discontinuation of therapeutic anticoagulation.

## Data Availability Statement

All datasets generated for this study are included in the article/Supplementary Material.

## Ethics Statement

The studies involving human participants were reviewed and approved by SUNY Downstate School of Medicine Institutional Review Board. The patients/participants provided their written informed consent to participate in this study. Written informed consent was obtained from the legal guardian/next of kin for the publication of any potentially identifiable images or data included in this article.

## Author Contributions

AE: case investigation, manuscript draft, and manuscript revisions. AD and KH: case discussion and manuscript revisions. GD: case discussion manuscript draft and manuscript revisions. All authors contributed to the article and approved the submitted version.

## Conflict of Interest

The authors declare that the research was conducted in the absence of any commercial or financial relationships that could be construed as a potential conflict of interest.
